# Estimation of phosphorus requirements of sows based on 24-h urinary phosphorus excretion during gestation and lactation

**DOI:** 10.1017/S0007114521003421

**Published:** 2022-08-14

**Authors:** Mariola Grez-Capdeville, Thomas D. Crenshaw

**Affiliations:** Department of Animal and Dairy Sciences, University of Wisconsin-Madison, Madison, WI 53706, USA

**Keywords:** Dose–response models, Phosphorus homoeostasis, Sow nutrition, Renal regulation, Oversupplementation

## Abstract

Phosphorus requirements of reproducing sows were estimated using 24-h urinary P excretion. Thirty-six multiparous sows were fed one of six maize–soybean meal-based diets with total P ranging from 0·40 to 0·80 % in 0·08 % increments with a constant Ca:total P ratio (1·25:1). Diets were fed from day 7·5 ± 1 after breeding until the end of lactation (day 26 ± 1). Urine samples were collected in mid and late gestation (days 77·1 ± 2 and 112·4 ± 1) and early and late lactation (days 4·5 ± 1 and 18·2 ± 1). Phosphorus requirements were estimated using linear and nonlinear regression models. Based on a single 24-h urinary P excretion, estimated daily dietary total P requirements in mid and late gestation were 10·3 g (6·0 g standardised total tract digestible P, STTD P), and estimates for early and late lactation were 31·1 g (16·6 g STTD P) and 40·3 g (22·1 g STTD P), respectively. Plasma P and Ca concentrations were maintained within normal ranges at the estimated levels of P requirements. No differences among treatments were observed for plasma parathyroid hormone (*P* ≥ 0·06) and bone formation marker (*P* ≥ 0·16). In lactation, bone resorption marker decreased (*P* ≤ 0·001) as sows consumed more P. Among the analysed variables, urinary P was the most sensitive response to changes in dietary P intake. Urinary P excretion offers a practical method to estimate P requirements in sows. Our recommended daily total P requirements are 10·3 g for gestation and 35·7 g for lactation.

Phosphorus (P) is involved in important physiological functions including energy metabolism, cell signaling, acid–base balance and the structural composition of skeletal tissue. Adequate P nutrition of reproducing sows is critical to maintain maternal P homoeostasis and skeletal integrity and to promote growth and development of their offspring^(1–3)^. Providing an optimal dietary P concentration is especially relevant as a major cause of sow mortality and premature culling involves lameness and locomotion problems^(4)^. On the other hand, P overfeeding represents an environmental concern. Dietary P supplemented in excess of requirements exceeds the ability of animals to absorb and retain P increasing the P content of manure. Excessive surplus nutrients, primarily P, from agricultural systems is one of the major anthropogenic factors associated with water eutrophication, which has drastic impacts in freshwater ecosystems^(5)^. The environmental consequences of oversupplementation of P demand that intensive animal operations improve the efficiency of P use. Therefore, precise information on nutritional P requirements is fundamental to formulate nutritionally adequate diets and to minimise the potential risk of water pollution from nonpoint-source P.

Empirical dose–response studies are a common approach to estimate P requirements of growing pigs. Conventional traits measured in response to incremental dietary P concentrations include growth, apparent P retention, bone mineralisation and bone strength parameters^(3,6–10)^. Except for growth, measurements of these traits require use of metabolic crates, postmortem collection of bones or use of whole-body imaging technologies. However, the financial value of the sow, complicated with needs for the maternal care of offspring and the large size of sows, make similar studies in sows financially infeasible and impractical to accomplish. Consequently, limited studies have been conducted to determine P requirements of sows during gestation and lactation. Current dietary recommendations for reproducing sows are primarily based on factorial modelling approaches^(11,12)^, which have not been validated with empirical data.

Phosphorus homoeostasis is maintained through physiological regulations of intestinal absorption from the diet, renal reabsorption and bone resorption^(13)^. The primary hormones involved in these processes include parathyroid hormone (PTH), 1,25-dihydroxyvitamin D and fibroblast growth factor-23 (FGF23)^(13)^. Although several additional endocrine signals and multiple organs are involved in maintaining P homoeostasis, the kidneys are ultimately responsible for P homoeostasis through adjustments in the amount of P reabsorbed or excreted via urine^(14)^. Numerous balance studies conducted with growing swine have associated urinary P excretion with dietary P intake^(15–17)^. Growing pigs and sows fed P-deficient diets excrete negligible amounts of P in urine^(6,18–20)^, referred to as endogenous excretion or inevitable P losses. In growing pigs, a minimal amount of urinary P is excreted until P intake is sufficient to restore levels of P in the body^(17)^. Therefore, the inflection point of dietary P intake at which urinary P increases above basal excretion may be considered as the dietary P level required to meet physiological needs. Due to the impracticality of using conventional response traits to determine P requirements in sows, the objective of this study was to evaluate the use of 24-h urinary P excretion as a relatively simple and practical alternative method.

## Material and methods

The animal portion of this experiment was conducted at the University of Wisconsin-Madison Swine Research and Teaching Center. All animal procedures were approved by the University of Wisconsin-Madison Institutional Animal Care and Use Committee (protocol A005638).

### Animals and experimental design

Thirty-six crossbred PIC Camborough sows (parity 3 to 7) were used in three blocks to estimate total P requirements in mid (day 77·1 ± 2) and late (day 112·4 ± 1) gestation and early (day 4·5 ± 1) and late (day 18·2 ± 1) lactation. In each block, twelve sows were randomly assigned to one of six dietary treatments (*n* 2 sows/diet/block). The study was initiated at day 7·5 ± 1 after breeding and was continued until day 35·5 ± 1 of the subsequent gestation period (next gestation). Sows were housed individually throughout gestation and lactation. In the first gestation-lactation period, pregnant sows were moved to farrowing crates at day 76·1 ± 2 of gestation to facilitate urine collections. Within 48 h after birth, litter size was standardised by cross-fostering to at least twelve pigs or up to the number of functional mammary glands. When necessary, piglets of the same age, but born from non-experimental sows were used to standardise litter size. After weaning (day 26·6 ± 1), sows were returned to gestation crates and bred using artificial insemination techniques upon display of estrous. Pregnancy was confirmed by ultrasound at day 30 after insemination. Sows were weighed the day before initiating the experiment, the day before urine sample collections and at weaning. Individual pig weights were recorded at birth, early lactation, late lactation, weaning and 3 weeks after weaning (post-weaning nursery phase).

### Dietary treatments and feeding management

Experimental diets (Table 1 and Table 2) were formulated to provide incremental concentrations of total P (0·40, 0·48, 0·56, 0·64, 0·72 and 0·80 %). Calcium:total P ratio was maintained constant at 1·25:1 across diets. Maize–soybean meal-based basal diets were formulated to meet or exceed the recommended requirements by the NRC 2012^(11)^ for gestation and lactation of all nutrients other than vitamin D, P and Ca. The vitamin D concentrations exceeded the NRC 1998^(21)^ minimum recommendations. Limited research evidence supports the use of the NRC 2012^(11)^ recommended or greater concentrations as reviewed by Amundson and Crenshaw^(22)^. Therefore, the experimental diets in this study were formulated to provide the same concentrations of vitamin D as routinely fed to sows in our research herd (8·125 ug cholecalciferol/kg diet). Samples of each batch of basal diet were collected and analysed to determine concentrations of P and Ca. Limestone and monocalcium phosphate were adjusted to provide the desired concentrations of total P and Ca in each dietary treatment. Sows were fed gestation diets (2·0 kg/d) from day 7·5 ± 1 until day 106 ± 1 of gestation and lactation diets from day 107 ± 1 of gestation until weaning. Lactation diets were restricted (2·0 kg/d) during the last days of gestation, and free access to feed was offered from the day of farrowing until weaning. Feed intake during lactation was determined in intervals from day 0 to 5, 5 to 12, 12 to 19 and 19 to weaning. Intervals from day 0 to 5 and day 12 to 19 were used to calculate average daily feed intake in early and late lactation, respectively. After weaning, all sows and pigs were fed the standard gestation (2·2 kg/d; 0·65 % Ca and 0·60 % total P) and post-weaning nursery diets, respectively, as routinely provided for animals at these production phases at the Swine Research and Teaching Center. Sows and pigs had free access to fresh water throughout the experimental period.

**Table 1.** Ingredients and nutrient composition of gestation diets (as-fed basis)*

	Total P, %					
	0·40	0·48	0·56	0·64	0·72	0·80
Ingredients, g/100 g diet						
Maize	83·66	83·13	82·61	82·08	81·55	81·03
Soybean meal, 48 % crude protein	11·74	11·78	11·83	11·87	11·92	11·96
l-Lysine mHCl	0·10	0·10	0·10	0·10	0·10	0·10
Monocalcium phosphate	0·39	0·78	1·16	1·55	1·93	2·32
Limestone	0·71	0·80	0·90	1·00	1·10	1·19
Max Fat†	1·00	1·00	1·00	1·00	1·00	1·00
NaCl	0·40	0·40	0·40	0·40	0·40	0·40
UW VTMM-S‡	2·00	2·00	2·00	2·00	2·00	2·00
Calculated nutrient content§						
Digestible energy, kcal/kg	3464	3448	3431	3414	3397	3380
Crude protein, %	12·6	12·6	12·6	12·5	12·5	12·5
Lysine, %	0·65	0·65	0·65	0·65	0·65	0·65
Ca, %	0·50	0·60	0·70	0·80	0·90	1·00
Total P, %	0·40	0·48	0·56	0·64	0·72	0·80
STTD P, %||	0·19	0·26	0·33	0·41	0·48	0·55
Analysed nutrient content¶						
Ca, %	0·56	0·69	0·76	0·85	0·97	1·04
Total P, %	0·41	0·48	0·56	0·65	0·73	0·80

* Diets were fed (2·0 kg/d) from day 7·5 ± 1 until day 106 ± 1 of gestation.

† Blend of animal and vegetable fats (Maxco Inc., Greenbay, WI, USA).

‡ University of Wisconsin vitamin and trace mineral mix-sow (UW VTMM-S) provided the following nutrients per kilogram of diet: retinol, 1680 ug; cholecalciferol, 8·125 ug; DL-*α*-tocopherol, 54 mg; menadione nicotinamide bisulphite, 0·75 mg; biotin, 0·4 mg; choline, 1350 mg; folic acid, 2·2 mg; nicotinamide, 22 mg; pantothenic acid, 24 mg; riboflavin, 11 mg; cyanocobalamin, 43 ug; Cu, 4 mg; I, 0·3 mg; Fe, 74; Mn, 22 mg; Se, 0·2 mg and Zn, 90 mg.

§ Calculated compositions from NRC^(21)^ values for feed ingredients.

|| Standardised total tract digestible P. Calculated from values for feed ingredients in NRC^(11)^.

¶ Analysed compositions. Values represent the mean of five to six batches.

**Table 2.** Ingredients and nutrient composition of lactation diets (as-fed basis)*

	Total P, %					
	0·40	0·48	0·56	0·64	0·72	0·80
Ingredients, g/100 g diet						
Maize	68·26	67·73	67·21	66·68	66·15	65·63
Soybean meal, 48 % crude protein	26·31	26·35	26·40	26·44	26·49	26·54
l-Lysine mHCl	0·10	0·10	0·10	0·10	0·10	0·10
Monocalcium phosphate	0·12	0·51	0·89	1·27	1·66	2·04
Limestone	0·71	0·81	0·90	1·00	1·10	1·20
Max Fat†	2·00	2·00	2·00	2·00	2·00	2·00
NaCl	0·50	0·50	0·50	0·50	0·50	0·50
UW VTMM-S‡	2·00	2·00	2·00	2·00	2·00	2·00
Calculated nutrient content§						
DE, kcal/kg	3541	3525	3508	3491	3474	3457
Crude protein, %	18·3	18·2	18·2	18·2	18·2	18·1
Lysine, %	1·05	1·05	1·05	1·05	1·05	1·05
Ca, %	0·50	0·60	0·70	0·80	0·90	1·00
Total P, %	0·40	0·48	0·56	0·64	0·72	0·80
STTD P, %||	0·18	0·25	0·32	0·39	0·46	0·53
Analysed nutrient content¶						
Ca, %	0·58	0·71	0·81	0·91	1·01	1·09
Total P, %	0·42	0·51	0·58	0·69	0·73	0·85

* Diets were fed from day 107 ± 1 of gestation until the end of lactation (day 26·6 ± 1).

† Blend of animal and vegetable fats (Maxco Inc., Greenbay, WI, USA).

‡ University of Wisconsin vitamin and trace mineral mix-sow (UW VTMM-S) provided the following nutrients per kilogram of diet: retinol, 1680 ug; cholecalciferol, 8·125 ug; DL-*α*-tocopherol, 54 mg; menadione nicotinamide bisulphite, 0·75 mg; biotin, 0·4 mg; choline, 1350 mg; folic acid, 2·2 mg; nicotinamide, 22 mg; pantothenic acid, 24 mg; riboflavin, 11 mg; cyanocobalamin, 43 ug; Cu, 4 mg; I, 0·3 mg; Fe, 74; Mn, 22 mg; Se, 0·2 mg; and Zn, 90 mg.

§ Calculated compositions from NRC^(21)^ values for feed ingredients.

|| Standardised total tract digestible P. Calculated from values for feed ingredients in NRC^(11)^.

¶ Analysed compositions. Values represent the mean of four to six batches.

### Urine collections

Total 24-h urine excretion was collected using Foley bladder catheters (18 or 20 Fr, 30 ml balloon) in mid and late gestation, early and late lactation and on day 35·5 ± 1 of the subsequent gestation. The peri-vulvar area was cleaned with a surgical scrub and a disinfectant solution (povidone-iodine 0·75 % titratable iodine) before catheter insertion. A sterile catheter coated with a topical triple antibiotic ointment (Neomycin-Bacitracin-Polymyxin) was introduced through the urethral opening into the bladder. The balloon was inflated with warm water and correct placement was confirmed by evidence of urine flow. The external end of the catheter was attached to a collection tube and urine was collected in buckets (20 l) containing 5 ml of a 10 % thymol-isopropanol solution as a preservative. After the 24-h collection period, the catheter was removed. Daily urine excretion (g/d) was calculated as the difference between bucket weight after the 24-h and empty bucket weight at time 0. Sample aliquots (50 ml) were collected and stored at 4°C until subsequent analysis of Ca and P.

### Blood collection and plasma analysis

Blood samples were collected into vacutainer tubes (6 ml, K_2_ EDTA 10·8 mg; BD Vacutainer) immediately after each 24-h urine collection. All blood samples were drawn between 07.00 and 09.00 hours. Gestating sows were sampled under fasted conditions. Blood samples were centrifuged for 20 min at 3000 *g*, and plasma aliquots were stored at −80°C until analysis.

Plasma concentrations of carboxyl-terminal propeptide of type I collagen (CICP) were measured as a marker of collagen synthesis and bone formation and carboxyl-terminal collagen type I crosslinks (CTX-I) as a marker of collagen degradation and bone resorption. Plasma CICP was measured using the MicroVue Bone CICP EIA kit (Cat. #8005; Quidel Corporation). The lowest detection limit was 0·20 ng/ml, and the determined inter- and intra-assay coefficients of variation were 10·4 % and 8·8 %, respectively. Plasma CTX-I was measured using the Serum CrossLaps carboxyl-terminal collagen type I crosslinks ELISA kit (Cat. #AC-02F1; Immunodiagnostic Systems Ltd., Boldon, Tyne & Wear, UK). The lowest detection limit was 0·02 ng/ml, and the determined inter- and intra-assay coefficients of variation were 4·7 % and 4·1 %, respectively. Plasma PTH was measured using Human BioActive PTH 1–84 ELISA kit (Cat. #60–3000; Immutopics Inc., San Clemente, CA, USA). The lowest limit of detection was 1·0 pg/ml, and the determined inter- and intra-assay coefficients of variation were 19·0 % and 12·6 %, respectively. All samples were analysed in duplicates.

### Phosphorus and calcium analysis in feed, urine and plasma samples

Feed (1·5 g) and urine (20 g) samples were digested with a nitric–perchloric acid mixture (3:1 v/v) using procedures previously described for our laboratory^(23)^. Concentrations of P in feed digests, urine digests and plasma samples were determined by spectrophotometry (Gilford Spectrophotometer 260, Gilford Instrument Laboratories, Inc.). The molybdovanadate method was used to determine P concentrations in feed and urine digests. Plasma P was determined using the molybdenum blue method after sample deproteinisation with trichloroacetic acid. Concentrations of Ca in plasma and feed digests were determined by flame atomic absorption spectrometry (Perkin-Elmer AAnalyst 400, Perkin-Elmer Corporation, Norwalk, CT). All samples were analysed in duplicates. Spike-recovery tests were used to evaluate accuracy of analytical methods. The spike recoveries for P and Ca analyses averaged 100 ± 8 % and 102 ± 8 %, respectively.

### Statistical analysis

Data were analysed as a completely randomised design using PROC MIXED of SAS (version 9.4; SAS Institute, Inc.). Individual sows were considered experimental units in all statistical analysis. Dietary total P concentration was used as a fixed effect and parity number as a random effect nested within block. Based on forward stepwise regression and backward elimination procedures, parity number was selected as an explanatory variable and block was eliminated and was not included in the final model. The model was fitted to allow heterogeneous variances between treatment groups. Orthogonal contrasts were used to determine linear (*P*-linear) and quadratic (*P*-quadratic) effects of dietary total P concentrations on sow and litter performance parameters, plasma measurements and urine P and Ca excretion. Values are reported as least squares means with pooled standard error of the means (sem). Dietary treatment effect was considered statistically significant at *P* ≤ 0·05 and a trend with 0·05 < *P* ≤ 0·08.

### Estimation of total phosphorus requirements

Three regression models were tested to determine dietary total P requirements based on urinary P excretion. Models included broken-line linear (plateau-linear), broken-line quadratic (plateau-quadratic) and four-parameter logistic models. The models were fitted to data within each individual day of urine collection in mid and late gestation and early and late lactation using PROC NLIN of SAS. The inverse of the variance was used as a weighting variable to correct heteroscedasticity^(24,25)^. Calculated adjusted coefficient of determination (adjusted-R^2^) and Akaike information criterion were used as model selection criteria^(26)^.

The plateau-linear and plateau-quadratic models were fitted to the adapted functions described by Robbins *et al.* ^(27)^.

Plateau-linear:

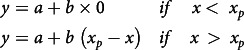




Plateau-quadratic:

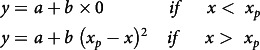




where 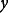 is the urinary P excretion (g/d), 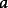 is the lowest urinary P excretion (plateau; g/d), 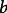 is the slope for the second segment, 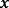 is the dietary total P concentration (%) and 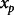 is the breakpoint (estimated total P requirement; %). The expressions 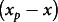 for the plateau-linear model and 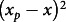 for the plateau-quadratic model are defined as zero when x < x_P_.

The four-parameter logistic model^(25)^ was fitted using the following function:

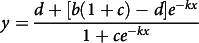

where 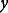 is the urinary P excretion (g/d), 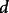 is the maximum urinary P excretion (g/d), 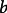 is the intercept for y axis, 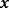 is the dietary total P concentration (%), 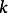 is a scaling parameter that scales x and 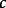 is a shaping parameter.

The estimation of total P requirement using the logistic model was derived from mathematical equations described by Kaps *et al.* ^(28)^ and Doepel *et al.* ^(29)^ (online Supplementary Material SM1). The second derivative of the logistic function was used to calculate the extreme points (minimum and maximum) in the curve of urinary P excretion. Estimated total P requirement was defined as the minimum extreme point, which represents the beginning of the acceleration phase of urinary P excretion. This point was calculated by equating the third-order derivative to zero. Estimated total P requirement ( 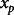 , %) was computed as:

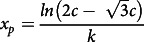




## Results

### Sow and litter performance parameters

Sow feed intake and performance parameters are reported primarily to establish the performance traits (Table 3). Effects of dietary P concentration on average daily feed intake, body weight (BW) and BW change were not detected (*P* ≥ 0·20). No feed refusal was observed in gestating sows. Feed intake in early lactation (6·1 ± 0·1 kg/d) was lower than in late lactation (7·6 ± 0·3 kg/d) and averaged 7·4 ± 0·1 kg/d for the total lactation period. Initial BW across dietary treatment groups was 225 ± 9 kg and increased as gestation progressed (234 ± 9 in mid and 255 ± 11 kg in late gestation), resulting in an average BW gain of 31 ± 5·6 kg during gestation. Body weight change during lactation was variable, ranging from −6·9 to 2·0 kg. However, BW at the next gestation was similar across treatments (243 ± 9 kg; *P* ≥ 0·71) and higher than initial BW.

**Table 3.** Feed intake, body weight, and change in weight of sows during gestation and lactation*

	Total P, %							*P*-value	
Items	0·40	0·48	0·56	0·64	0·72	0·80	sem	Linear	Quadratic
Number of sows	6	5	6	6	5	6			
ADFI, kg/d†									
Early lactation	5·7	6·2	6·3	5·8	6·2	6·6	0·33	0·20	0·74
Late lactation	7·6	8·2	7·6	7·8	6·9	7·7	0·45	0·37	0·99
Entire period	7·2	7·3	7·6	7·5	7·1	7·6	0·28	0·65	0·71
BW, kg									
Initial‡	232	219	229	217	225	225	10·8	0·62	0·35
Mid gestation	242	226	238	233	230	238	12·2	0·88	0·47
Late gestation	262	256	258	249	252	255	14·2	0·49	0·57
Early lactation	251	242	252	243	250	239	12·2	0·57	0·72
Late lactation	247	245	253	243	244	239	12·2	0·46	0·56
Next gestation§	247	240	242	241	251	237	11·2	0·71	0·99
Weight change, kg									
Gestation||	30·0	37·1	28·7	32·8	27·2	30·2	7·99	0·63	0·86
Lactation¶	–6·9	2·0	–2·0	0·6	–0·4	1·3	3·99	0·28	0·49

ADFI, average daily feed intake; BW, body weight.

* Values represent the least squares means. Parity number was used as a covariate. Data were collected on days 77·1 ± 2 (mid gestation) and 112·4 ± 1 (late gestation) of gestation, and days 4·5 ± 1 (early lactation) and 18·2 ± 1 (late lactation) of lactation.

† Intervals from day 0 to 5, day 12 to 19 and day 0 to weaning (26·6 ± 1 d) were used to calculate ADFI in early lactation, late lactation and over the entire lactation period.

‡ BW at day 6·5 ± 1 after breeding (day before starting dietary treatments).

§ BW at day 34·5 ± 1 of the subsequent gestation period.

|| Weight difference between initial and late gestation BW.

¶ Weight difference between early lactation and weaning BW.

Litter size, weight and average daily gain (ADG) in lactation were not different among treatments (*P* ≥ 0·15; Table 4). Initial litter size (after cross-fostering) was 15·1 ± 0·4 pigs, and 12·5 ± 0·4 pigs per litter were weaned. Litter ADG during the total lactation period was 2·9 ± 0·1 kg (0·234 ± 0·01 kg/d/pig). Three weeks after the post-weaning nursery phase, the number of pigs was similar across treatments (12·4 ± 0·4; *P* ≥ 0·48). However, there was a linear increase (*P* ≤ 0·03) in pig BW (from 12·4 to 14·2 kg) and ADG (from 0·248 to 0·308 kg/d) in response to maternal total P concentration in gestation and lactation diets.

**Table 4.** Effect of maternal dietary phosphorus concentrations on offspring performance parameters*

	Total P, %†							*P*-value	
Items	0·40	0·48	0·56	0·64	0·72	0·80	sem	Linear	Quadratic
Litter size, *n*									
Born alive‡	14·0	14·8	14·6	14·3	12·7	15·1	1·08	0·90	0·81
After CF§	15·3	15·4	16·0	13·9	14·6	15·5	0·66	0·50	0·41
Weaning	13·3	12·0	12·2	12·5	12·5	12·5	0·79	0·71	0·42
Litter weight, kg									
Birth‡	18·7	19·8	18·6	19·2	14·5	21·3	2·06	0·89	0·33
Early lactation	27·3	23·4	25·4	25·7	23·8	28·6	2·15	0·65	0·15
Late lactation	69·9	63·9	69·5	68·2	63·9	71·2	4·15	0·89	0·48
Weaning	94·1	91·7	99·0	94·9	87·8	97·5	5·81	0·98	0·96
Litter ADG, kg/d									
Early lactation	1·8	1·6	1·7	1·7	1·7	1·8	0·17	0·85	0·53
Late lactation	3·1	3·0	3·2	3·2	2·9	3·2	0·18	0·94	0·84
Entire period||	2·8	2·9	3·1	2·9	2·8	2·9	0·15	0·84	0·41
Post-weaning nursery phase									
Pigs, *n*	13·1	11·9	12·2	12·5	12·5	12·5	0·79	0·87	0·48
BW, kg	12·4	13·5	13·9	13·5	13·3	14·2	0·50	0·03	0·38
ADG, g	248	273	276	281	301	308	21·78	0·02	0·90

CF, cross-fostering; ADG, average daily gain; BW, body weight.

* Values represent the least squares means of 4 to 6 sows/treatment. Parity number was used as a covariate. Litter data were collected on days 4·5 ± 1 (early lactation) and 18·2 ± 1 (late lactation) of lactation. Measurements for the nursery phase were collected 3 weeks after weaning. All pigs were fed with the same standard diet during the post-weaning nursery phase.

† Total phosphorus concentrations in diets fed to sows during gestation and lactation.

‡ Means do not include cross-fostered pigs.

§ Litter size was standardised within 48 h after farrowing to maximise the use of functional mammary glands.

|| ADG over the entire lactation period (birth to day 26·6 ± 1).

### Total 24-h urine phosphorus and calcium excretion

**Fig. 1.**
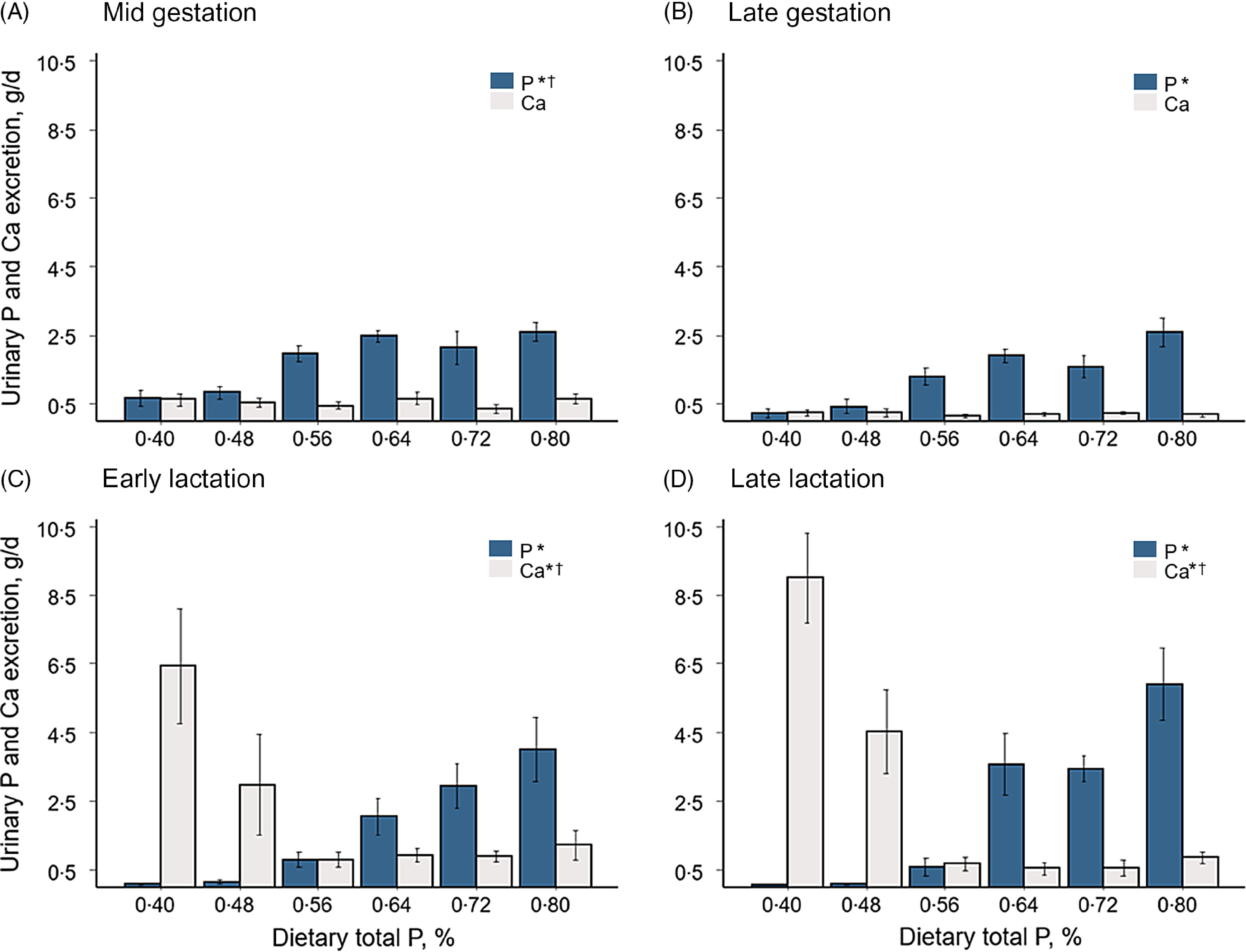
Urinary P and Ca excretion of sows fed incremental concentrations of total P. Complete 24-h urine excretion was collected in mid (day 77·1 ± 2) and late (day 112·4 ± 1) gestation and early (day 4·5 ± 1) and late (18·2 ± 1) lactation. Values represent the least squares means of 4 to 6 sows/treatment ± sem. Parity number was used as a covariate. *P-linear < 0·001; ^†^ *P*-quadratic ≤ 0·01.

The amount of urine excreted for each of the total 24-h collections was not significantly affected by dietary treatments (online Supplementary Table S1). Daily excretions of urinary P and Ca in gestation and lactation are shown in Fig. 1. Excretion of P in urine increased with higher intakes of total P in mid (*P*-linear < 0·001, *P*-quadratic = 0·01) and late gestation (*P*-linear < 0·001). However, urinary Ca was not affected by the concentration of total P in the diet. Although there was a similar pattern of P and Ca excretion between the two evaluated stages of gestation, urinary P and Ca were greater in mid gestation (1·78 ± 0·18 g P/d; 0·54 ± 0·10 g Ca/d) than in late gestation (1·33 ± 0·16 g P/d; 0·20 ± 0·04 g Ca/d).

**Fig. 2.**
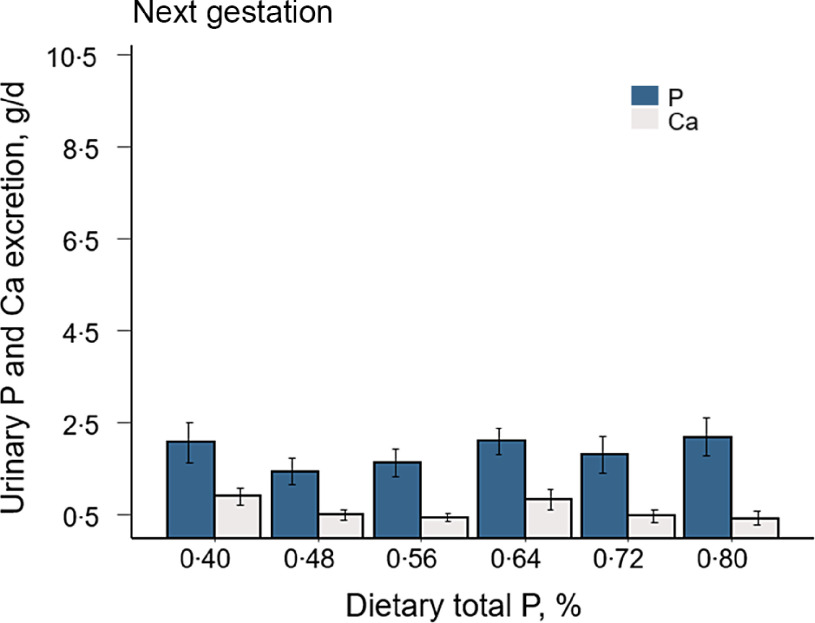
Urinary P and Ca excretion in the next gestation. All sows were fed the same concentrations of total P (0·60 %) during the next gestation period. Dietary total P represents the concentrations of P in the previous gestation and lactation diets. Complete 24-h urine excretion was collected at day 35·5 ± 1 of gestation. Values represent the least squares means of 4 to 6 sows/treatment ± sem. Parity number was used as a covariate. P: *P*-linear = 0·46, *P*-quadratic = 0·29; Ca: *P*-linear = 0·11, *P*-quadratic = 0·73.

In early and late lactation urinary P increased (*P*-linear< 0·001) and urinary Ca decreased (*P*-linear = 0·003, *P*-quadratic = 0·005) with higher concentrations of P in the diet. Sows fed the two lowest concentrations of total P (0·40 and 0·48 %) excreted marginal amounts of P in urine (0·11 ± 0·10 g/d in early and 0·08 ± 0·05 g/d in late lactation). Urinary P increased in sows fed total P concentrations greater than 0·48 %, reaching an excretion of 4·01 ± 0·93 g P/d in early and 5·91 ± 1·05 g P/d in late lactation in sows fed 0·80 % total P. The greatest urinary Ca excretion was observed in sows fed the lowest concentration of P (6·44 ± 1·67 g/d in early and 9·01 ± 1·32 g/d in late lactation). Urinary Ca excretion decreased with increasing dietary total P up to 0·56 % and remained constant after this level (0·88 ± 0·63 g/d in early and 0·63 ± 0·40 g/d in late gestation). Excretions of P (1·86 ± 0·25 g/d) and Ca (0·59 ± 0·09 g/d) in urine at the next gestation were not affected by total P concentrations in the diets fed during the previous gestation and lactation periods (Fig. 2).

### Estimation of total phosphorus requirements

**Fig. 3.**
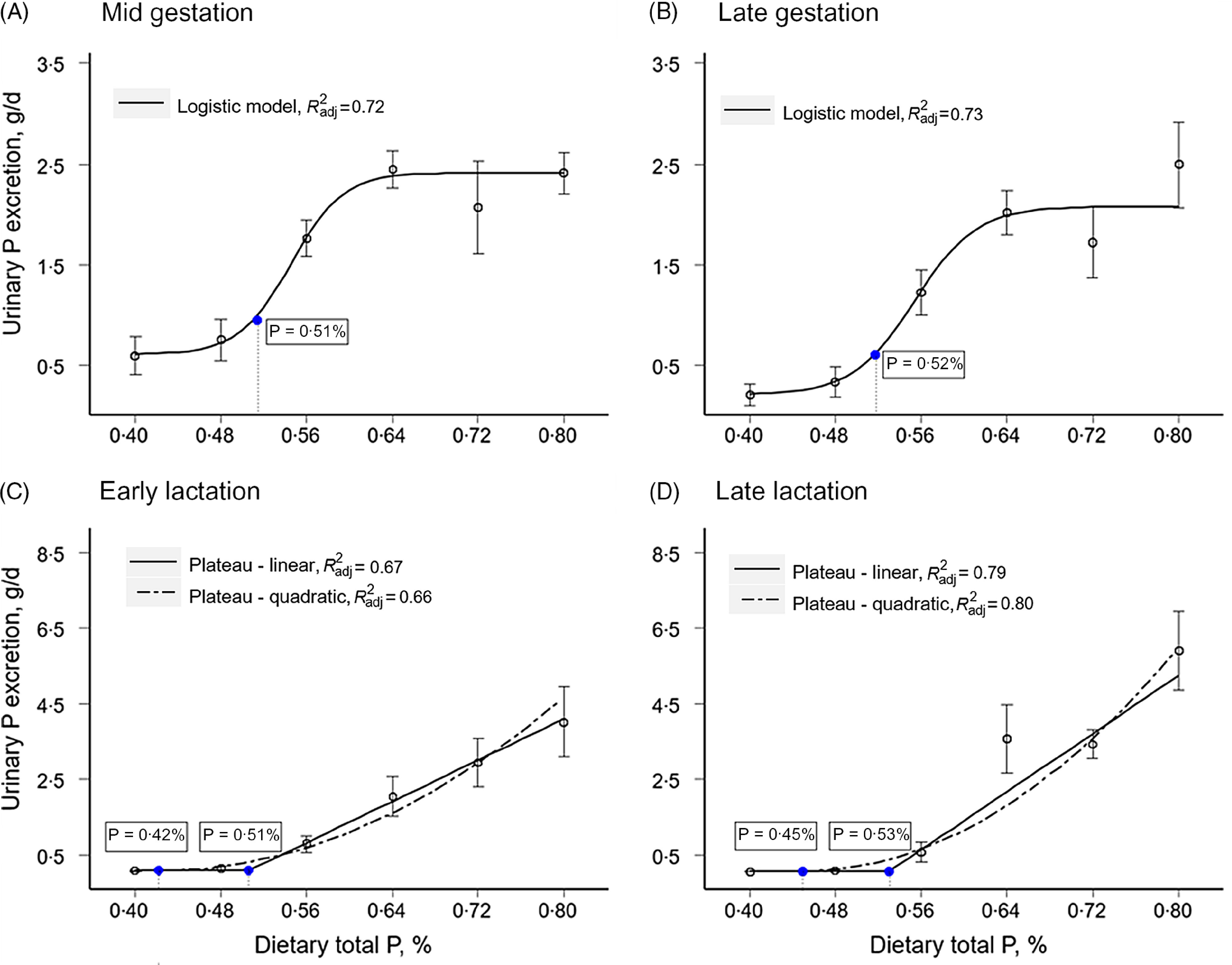
Estimation of dietary total P requirements based on 24-h urinary P excretion in mid and late gestation (days 77·1 ± 2 and 112·4 ± 1) and early and late lactation (days 4·5 ± 1 and 18·2 ± 1). Open symbols (○) represent means of 4 to 6 sows/treatment ± sem. Filled symbols (●) and values in the boxes represent the estimated total P requirements using the logistic, plateau-linear, and plateau-quadratic models, respectively. *R* ^2^_adj_, adjusted coefficient of determination.

Based on adjusted-*R* ^2^ and Akaike information criterion (online Supplementary Table S2), selected regression models to determine total P requirements were the logistic model for gestating sows and the plateau-linear and plateau-quadratic models for lactating sows (Fig. 3). Estimated total P requirements using the logistic model were 0·51 % (10·2 g/d) in mid gestation and 0·52 % (10·4 g/d) in late gestation. Estimated total P requirements in lactating sows using the plateau-linear model were approximately 20 % greater than estimated values with the plateau-quadratic. In early lactation, estimated total P with the plateau-linear model was 0·51 % (95 % CI 0·46, 0·55 %) and 0·42 % (95 % CI 0·38, 0·46 %) with the plateau-quadratic model. Calculated total P intake at these concentrations was 31·1 and 25·6 g/d, respectively. In late lactation, total P requirements were higher than in early lactation. The estimated value with the plateau-linear model was 0·53 % (95 % CI 0·50, 0·57 %) and 0·45 % (95 % CI 0·43, 0·47 %) using the plateau-quadratic model, corresponding to 40·3 and 34·2 g/d, respectively.

### Plasma concentrations of phosphorus, calcium, parathyroid hormone and bone turnover markers

Plasma measurements are shown in Table 5. Plasma concentrations of P in mid gestation and Ca in late gestation linearly decreased (*P* = 0·04) with increasing P intake. Despite these specific responses, gestating sows maintained plasma concentrations of P and Ca within a narrow range (5·0 to 5·7 mg P/dl and 9·5 to 10·6 mg Ca/dl). During early lactation, plasma P concentrations increased with dietary P (*P*-linear and *P*-quadratic ≤ 0·004). In contrast, concentrations of Ca linearly decreased with increasing P intake (*P* = 0·003). This response persisted and was accentuated as lactation progressed. During late lactation, wider ranges of plasma P and Ca concentrations were observed (2·9 to 6·4 mg P/dl and 10·6 to 12·1 mg Ca/dl). However, clinical signs of P or Ca homeostatic disorders were not observed throughout the experiment, and plasma P and Ca concentrations at the next gestation were within expected physiological ranges and were not different across treatments (*P* ≥ 0·29).

**Table 5.** Effect of dietary total phosphorus concentration on plasma measurements in gestating and lactating sows*

		Total P, %							*P*-value	
	Period	0·40	0·48	0·56	0·64	0·72	0·80	sem	Linear	Quadratic
P, mg/dl	Mid gest	5·5	5·3	5·5	5·0	5·4	5·1	0·15	0·04	0·75
	Late gest	5·3	5·5	5·7	5·3	5·4	5·7	0·23	0·42	0·97
	Early lact	3·5	5·3	5·8	5·9	5·9	6·4	0·29	<0·001	0·004
	Late lact	2·9	4·8	5·2	6·0	5·6	6·4	0·29	<0·001	0·003
	Next gest	5·4	5·5	5·6	5·3	5·6	5·7	0·19	0·29	0·58
Ca, mg/dl	Mid gest	10·6	10·4	10·6	10·3	10·4	10·6	0·18	0·82	0·29
	Late gest	10·0	9·6	9·8	9·5	9·5	9·7	0·14	0·04	0·09
	Early lact	11·5	11·1	10·5	10·7	10·1	10·5	0·32	0·003	0·13
	Late lact	12·1	11·5	10·7	11·0	10·6	10·7	0·33	<0·001	0·04
	Next gest	9·7	9·8	9·8	9·9	9·8	9·9	0·11	0·29	0·65
PTH, pg/ml	Mid gest	35·5	10·4	12·8	17·7	13·9	4·7	7·66	0·06	0·47
	Late gest	35·4	12·6	27·5	26·3	61·6	27·2	15·95	0·41	0·86
	Early lact	17·3	2·6	15·1	16·7	26·2	9·0	5·92	0·57	0·68
	Late lact	12·1	4·4	17·9	11·5	25·6	11·8	5·01	0·22	0·55
CTX-I, ng/ml	Mid gest	1·1	1·0	1·3	0·9	0·9	1·2	0·13	0·70	0·53
	Late gest	1·8	1·5	1·6	1·2	1·4	1·6	0·18	0·20	0·08
	Early lact	1·4	1·1	1·0	0·7	0·8	0·7	0·15	0·001	0·10
	Late lact	1·9	1·2	1·2	0·7	0·7	0·7	0·21	<0·001	0·03
CICP, ng/ml	Mid gest	74·5	367·8	102·8	75·1	32·7	40·3	122·86	0·22	0·59
	Late gest	59·7	118·7	63·3	55·3	41·5	36·8	32·26	0·16	0·56
	Early lact	56·7	91·0	58·4	55·6	45·2	39·3	23·23	0·22	0·56
	Late lact	48·2	48·4	42·3	45·9	39·8	33·9	9·65	0·25	0·71
CICP:CTX-I	Mid gest	70·5	409·9	92·5	82·1	37·1	39·4	136·37	0·24	0·60
	Late gest	35·0	89·7	44·3	48·3	28·6	25·8	25·69	0·25	0·37
	Early lact	45·7	93·8	66·7	83·8	49·7	56·7	24·53	0·77	0·28
	Late lact	28·3	41·8	36·3	68·1	51·2	55·1	11·81	0·06	0·37

gest, gestation; lact, lactation; PTH, parathyroid hormone; CTX-I, carboxyl-terminal collagen type I crosslinks; CICP, carboxyl-terminal propeptide of type I collagen; CICP:CTX-I, CICP:CTX-I ratio.

* Values represent the least squares means of 4 to 6 sows/treatment. Parity number was used as a covariate. Data were collected on days 77·1 ± 2 (mid gestation) and 112·4 ± 1 (late gestation) of gestation, days 4·5 ± 1 (early lactation) and 18·2 ± 1 (late lactation) of lactation and day 35·5 ± 1 of the subsequent gestation (next gestation).

In mid gestation, plasma PTH tended to decrease (*P*-linear = 0·06) with increasing P intake. Other than this response, no differences (*P* ≥ 0·16) among treatments were observed for plasma PTH and CICP concentrations at other collection times in gestation and during lactation. Circulating concentrations of carboxyl-terminal collagen type I crosslinks during gestation were not affected by dietary total P concentration (*P* ≥ 0·20). However, plasma CTX-I decreased with increasing P intake in early (*P*-linear = 0·001) and late lactation (*P*-linear < 0·001 and *P*-quadratic = 0·03). In late lactation, the CICP:CTX-I ratio tended to increase with P intake (*P* = 0·06).

## Discussion

### Urinary phosphorus and calcium excretion

The importance of renal handling of P in the maintenance of P homoeostasis was evidenced by changes in urinary P excretion associated with consumption of diets with different P concentrations. Renal regulation of P occurs through adjustments by tubular P reabsorption to maintain serum concentrations within the normal physiological range. Therefore, the P content in urine reflects P intake and post-absorptive utilisation of P^(15)^. Urinary P excretion in gestating (0·40 g/d) and lactating sows (0·07 g/d) fed low P diets (0·40 % total P) was minimal. Values of approximately 0·2 g/d were previously reported for unavoidable P losses in reproducing sows^(20,30)^. Similar to responses observed in growing pigs^(17)^, the excretion of P in urine was low and constant until the concentration of P in the diet approached optimum levels, which were assumed to have met soft tissue needs. After this point, lactating sows had a marked and continuous increase in urinary P as a function of P intake. However, during gestation, urinary P excretion tended to plateau in response to the higher concentrations of dietary P. An explanation for the plateau in responses observed in gestating sows might involve either physiological adaptations in intestinal P absorption or an increased retention in bone. According to Rodehutscord^(17)^, the relationship between P intake and P absorption is not linear and adjustments in P absorption occur depending on post-absorptive tissue needs. However, as physiological pathways for downregulation of P absorption in gestating sows fed excess P are not clearly established, the more likely explanation for the plateau in urinary P excretion relates to an increase in P accumulation in bone tissues. In the current experiment, fecal excreta were not collected and bone mineral accumulation was not measured, thus estimates of P absorption or retention were not available.

Urinary Ca excretion in gestation was constant and independent of dietary P levels. During lactation, sows fed low concentrations of P (0·40 to 0·48 % total P) excreted high amounts of Ca in the urine (8 to 24% of Ca intake). This response is consistent with previous human and animal studies^(31–33)^. In growing pigs, excessive urinary Ca losses, reduced apparent Ca retention and reduced bone mineralisation were associated with P-deficient diets independent of the level of Ca intake^(15,16,34,35)^. Therefore, the hypercalciuric effect of P depletion has been associated with insufficient P for adequate retention of absorbed Ca in bone^(35)^. The changes in urinary P in response to P intake indicate that a dietary Ca:P ratio of 1·25 may have been too wide when feeding P-deficient diets. Under limited P conditions, a more narrow dietary Ca:P ratio may favour Ca retention efficiency, as well as improve the efficiency of dietary P utilisation^(11)^. The changes in urinary Ca excretion in response to P intake underscore the close relationship between P and Ca metabolism. In addition to P excretion, measurements of urinary Ca and, more specifically, the Ca to P ratio may provide a useful approach to evaluate P status in sows.

### Estimation of total phosphorus requirements

Adequate dietary P supplied to gestating and lactating sows is important to meet requirements of P for soft tissue maintenance, maternal skeletal tissue deposition, accretion of P in the products of conception and milk production. Current recommendations of dietary P for sows are based on factorial modelling, which takes into account these multiple variables that contribute to the requirements^(11,12)^. Although factorial methods are widely used to estimate the optimal dietary nutrient supply, predictions of P requirements for sows are based on only a few studies. Empirical studies are needed to validate recommendations derived from factorial models to assure the formulation of nutritionally adequate diets and to avoid oversupplementation of P.

P requirements are commonly expressed on a total, available or digestible basis^(11,12)^. The formulation of swine diets using digestible values instead of total P has been promoted to ensure adequate P supply and to reduce excessive P excretion into manure^(6,36)^. Values of standardised total tract digestible (STTD) P for feedstuffs commonly used in swine diets have been reported by the NRC^(11)^. The vast majority of these values were determined in studies with growing and finishing pigs, which were extrapolated to values for sows. However, studies with sows have shown that digestibility of P and Ca is not constant but rather proportional to physiological stage and nutritional demand^(37,38)^. Therefore, as the digestibility values of feed ingredients for sows are unreliable and variable, we have expressed the estimated requirements based on total P, which allowed verification by laboratory analysis of the diets. Values of STTD P were calculated, using the reference values, to allow comparisons of the results obtained in this study with previously published literature.

In the current study, average daily feed intake during lactation (7·4 ± 0·1 kg) was greater than feed intake values (6·6 kg) assumed by the NRC^(11)^ to predict requirements of dietary P concentrations for lactating sows. As the amount of nutrient intake is a function of nutrient concentration in the diet and feed intake, requirements of dietary P were expressed as daily amounts (g/d).

### Gestation

The selection of the regression model to estimate optimal nutrient levels depends on the data set and dose–response relationship. The evaluation of different models is an effective approach to identify the model that best fits the data and offers an appropriate description of the biological response^(39)^. In this study, the logistic model provided the best fit for the urinary P excretion data in the two evaluated phases of gestation. The estimated values for total P requirements for sows in mid (day 77) and late (day 112) gestation were essentially the same (∼ 10·3 g total P/d; ∼ 6 g STTD P/d). This is consistent with previous researchers who recommended feeding sows a constant amount of dietary P from day 70 until the end of gestation^(12)^. Maternal mineral demand increases as gestation progresses due to the increased mineral accretion in the developing fetuses during the late gestation period^(40)^. To promote a more efficient use of nutrients, dietary guidelines suggest to phase-feed sows adjusting dietary nutrient levels by stages of gestation (early and late gestation). Data in this study were collected during the last month of gestation. If phase feeding is implemented, P requirements during the early gestation phase (< day 75) should be evaluated.

Estimated P requirements in this study align with the findings of Everts *et al.* ^(20)^, who estimated a daily minimal requirement of digestible P of 6 g during late gestation for pregnant sows evaluated over three parities. However, these values are below the recommended levels (∼ 7·5 g STTD P/d) based on factorial modelling by the NRC^(11)^ (recommendations for days of gestation> 90) and Bikker and Block^(12)^. Diverse productive and reproductive factors, such as parity, litter size and weight gain of nursing pigs, contribute to maternal nutritional requirements as these factors influence the amount of nutrients transferred to the gravid uterus, milk yield and maternal retention^(40–42)^. The differences between empirical results and factorial models may be attributed to the observed performance and the assumed performance values used in different modelling scenarios. Additional differences may involve inclusion of safety margins to compensate for animal variability and to correct for assumed values of P digestibility in feedstuffs^(20,36)^.

### Lactation

During lactation, the plateau-linear and plateau-quadratic models provided better fits for the data than logistic models. However, the estimated P requirements using the plateau-quadratic model were approximately 20 % below estimates derived from parameters using the plateau-linear model. These different results illustrate the concerns associated with the fact that estimates of optimal nutrient levels from dose–response data are dependent on the selected regression model^(27,39,43)^. Thus, the selection of a statistical model that explains biological responses is critical. In the current results, the estimated P requirement based on use of the plateau-quadratic model fell between the two lowest concentrations of P in the dietary treatment groups. Biological support to use the plateau-linear model, as discussed before, involved observations of urinary Ca excretion. Sows fed low P diets had excessive excretion of Ca in the urine, which has been associated with P-deficient diets. Therefore, we selected the plateau-linear model to predict the P requirements of sows during lactation.

Estimated P requirements in early lactation (31·1 g total P/d; 16·6 g STTD P/d) were lower than in late lactation (40·3 g total P/d; 22·1 g STTD P/d). These results are likely associated with a higher P demand as lactation progressed to support the requirements for milk production^(44)^. Although P demand is not constant throughout lactation, in practice sows are fed the same concentration of dietary P during the entire lactation phase. Consequently, researchers and dietary guidelines recommend one level of dietary P for lactating sows.

NRC^(11)^ recommendations for dietary P for multiparous lactating sows range from 18·9 to 24·0 g STTD P/d (34·1 to 40·8 g total P/d). These values were supported by Bikker and Block^(12)^ who predicted a requirement of 19·6 to 20·6 g STTD P/d and our estimates for late lactation. On the other hand, Everts *et al.* ^(30)^ analysed the requirements of lactating sows over three parities and recommended a digestible P intake of 16·5 g/d for sows with a litter ADG of 2·5 kg (litter size = 10·4 pigs). Although this value included a safety margin of 25 %, our estimated value in late lactation was still greater than their recommendations. Discrepancies might be related to the greater litter size (12·5 ± 0·4 weaned pigs) and litter ADG (2·9 ± 0·1 kg/d), and selected days of lactation used to estimate the requirements. In the current study, measurements in late lactation were performed at day 18, which represents the day of peak milk production^(44)^. In order to optimise P use by sows, dietary P recommendations should be representative of the requirements for the entire lactation period rather than a predicted level at maximum demand. Moreover, homoeostatic regulations of mineral metabolism that allow maternal adaptation to changes in dietary nutrient content and physiological needs are important considerations. As reviewed by Kovacs^(45)^, studies with humans and rodents have shown that maternal depletion of skeletal reserves to meet mineral demand during gestation and lactation is recovered after weaning. Similarly, Giesemann *et al.* ^(38)^ reported that bone loss in primiparous and multiparous sows during lactation was recovered during the next gestation period. The dynamic processes in bone and mineral metabolism allow a more efficient use of minerals during gestation and lactation, which should be considered in estimates of requirements for reproducing sows. In this study, estimates based on urinary excretions reflect the post-absorptive tissue needs without allowances made for depletion of some skeletal mineral reserves during the lactation phase. Therefore, our recommendation for dietary P for the entire lactation period is 35·7 g total P/d (19 g STTD P/d), which was calculated as the average between the estimates in early and late gestation. This value represents approximately 89 % of the estimated requirements in late lactation.

### Offspring performance

Litter size, weight and ADG during lactation were not associated with maternal dietary P concentrations. Given the small sample size, definitive conclusions on the association between maternal dietary P intake with reproductive and litter performance cannot be drawn from this study. Surprisingly, BW and ADG of pigs during the post-weaning nursery phase were affected by maternal P intake. Long-term effects of dietary P intake by reproducing sows on their offspring performance require further evaluation.

### Plasma phosphorus, calcium, parathyroid hormone and bone turnover markers

The analysis of plasma P and Ca concentrations and bone turnover markers indicated that mineral and bone metabolism was not altered in sows fed diets that provided the estimated requirements based on urinary P excretion. With the exception of lactating sows fed the lowest level of dietary P (0·40 %), plasma concentrations of P and Ca were maintained within the range considered normal for sows^(46)^. Homoeostasis of P was only disrupted under extreme conditions of low P intake during phases of high P demand in early and late lactation. Because of the important roles of P and Ca in multiple biological processes, circulating concentrations of these minerals are maintained at constant concentrations. Therefore, findings in this study are in agreement with those of other researchers who concluded that plasma P is a poor indicator of P status in swine^(3,15)^.

As the maintenance of P and Ca homoeostasis can result in a depletion of bone mineralisation, bone turnover markers provide relevant information on the association between nutritional P and Ca status and bone metabolic activity^(47)^. By-products of collagen type I synthesis (CICP) and degradation (CTX-I) have been used as serum markers to evaluate bone formation and resorption, respectively^(47,48)^. In this study, plasma CICP concentrations were unaffected by dietary P concentrations. However, on average across treatment groups, plasma CICP decreased as gestation and lactation progressed. This is consistent with the increased demand of P and Ca in late gestation and lactation, which may result in a reduction of maternal bone mineral deposition^(38)^. In lactation, dietary P concentrations levels were inversely associated with plasma CTX-I concentrations. Additionally, the ratio between CICP to CTX-I tended to increase with P intake in late lactation. These results imply that lactating sows fed low P diets mobilised P and Ca from skeletal reserves to achieve P and Ca homoeostasis as reflected by increased CTX-I concentrations. Despite this response, sows fed the lowest concentration of P failed to maintain plasma P within the normal range.

Based on a single blood sample collection, we found no association between plasma PTH and dietary P concentrations. The secretion of PTH, as well as FGF23, is positively stimulated by high P intake and hyperphosphataemia^(49–51)^. These hormones act on kidneys to decrease tubular P reabsorption and, therefore, maintain serum P constant through changes in urinary P excretion. PTH is also an important calcitropic hormone secreted by the parathyroid glands when serum Ca is low. The physiological function of PTH is to restore serum Ca levels by increasing renal Ca reabsorption and bone resorption. Therefore, observed changes in urinary P, urinary Ca and bone markers would be expected to be associated with plasma PTH concentrations. However, concentrations of dietary Ca were increased with changes in dietary P to maintain a constant Ca to P ratio across diets. Possibly, the level at which urinary P excretion increased above minimal renal excretion, used to estimate P requirements, corresponded with adequate dietary Ca. Thus, Ca intake was sufficient to support Ca homoeostasis which resulted in unaltered concentrations of plasma PTH. On the other hand, studies in human and rodent models have reported the critical role of FGF23 in the regulation of P homoeostasis^(13)^. The pronounced increases in urinary P as sows consumed more P might be explained by the phosphaturic action of FGF23. However, to our knowledge, validated assays to measure circulating concentrations of FGF23 in pigs are not available. Therefore, plasma FGF23 measurements were not considered in this study. Future research on FGF23 should be undertaken to better understand the role of this hormone in P metabolism in swine and to evaluate its potential use as a biological marker for P status.

### Conclusion

To our knowledge, this is the first study to empirically determine P requirements of prolific sows. Excretion of P in urine reflects dietary P intake and fluctuations in physiological P demand associated with different phases of gestation and lactation. Urinary P represents a more sensitive response variable to dietary P supply than plasma P concentrations and evaluated bone turnover markers. Modelling of the P intake – urinary P excretion curve provided reliable estimates of optimal P intake of reproducing sows. The values of daily total P requirements for sows based on a single 24-h collection were 10·2 g in mid gestation, 10·4 g in late gestation, 31·1 g in early lactation and 40·3 g in late lactation. On the basis of these findings, our recommendations of daily total P requirements of multiparous sows are 10·3 g (6 g STTD P) and 35·7 g (19 g STTD P) for gestation and lactation, respectively. These recommendations for P requirements of gestating sows are below previous estimates. However, estimates for lactating sows are surprisingly consistent with results from modelling of P requirements. Results in this study indicate that levels of total P in diets fed to gestating and lactating sows should not exceed recommendations reported by current dietary guidelines.

## Supplementary material

For supplementary material accompanying this paper visit https://doi.org/10.1017/S0007114521003421. click here to view supplementary material
