# Supplementary material for: Estimation of phosphorus requirements of sows based on 24-h urinary phosphorus excretion during gestation and lactation
Source: Br J Nutr. 2021 Sep 6;128(3):377–88. doi: 10.1017/S0007114521003421 (PMC9340856; doi:10.1017/S0007114521003421)
Supplement: Supplementary file 1 [file S0007114521003421sup001.docx]

### Supplementary material

## Estimation of phosphorus requirements of sows based on 24-hour urinary phosphorus excretion during gestation and lactation

Mariola Grez-Capdeville^1^ and Thomas D. Crenshaw^1^*

^1^Department of Animal and Dairy Sciences, University of Wisconsin-Madison, Madison, WI 53706, USA.

*Corresponding author: Mariola Grez-Capdeville. E-mail: [grezcapdevil@wisc.edu](mailto:grezcapdevil@wisc.edu)

*Supplementary Material SM1*: Estimation of total P requirements using the four-parameter logistic regression model (Figure 1).

Total 24-hour urinary P excretion (g/day) was fitted to the following logistic equation ^(1)^:

(1) Logistic function

$$y = \frac{d + \left[ b \left( 1 + c \right) - d \right] e^{-kx}}{1+c e^{-kx}}$$

Where $y$ is the urinary P excretion (g/day), $d$ is the maximum urinary P excretion (g/day), $b$ is the intercept for y axis, $x$ is the dietary total P concentration (%), $k$ is a scaling parameter that scales x, and $c$ is a shaping parameter.

The second-order derivative of the logistic function was calculated to determine the two extreme critical points, lower (LCP) and upper (UCP), in the logistic curve.

(2) Second-order derivative

$$y'' = -\frac{\left( c + 1 \right) \left( d - b \right)k^{2} e^{kx} ( e^{kx} - c)}{{( e^{kx} + c)}^{3}}$$

In the logistic curve, the LCP represents the beginning of the exponential phase. At this point, the change of the dependent variable value is accelerated in response to the independent variable. In contrast, the UPC represents the deceleration point. Values of the dependent variable at these critical points are calculated by equating the third-order derivative to zero.

(3) Third-order derivative

$$y''' = \frac{(c + 1) {\left( d - b \right)k}^{3} e^{kx} ( e^{2kx} -4ce^{kx}+ c^{2})}{{( e^{kx} + c)}^{4}}$$

(4) Zeros of the third-order derivative

$$UCP= \frac{\ln(\sqrt{3}c+2c)}{k}$$

$$LCP= \frac{\ln(2c- \sqrt{3}c)}{k}$$

In this experiment, the LCP was defined as the estimated total P requirement. At this point, the urinary P excretion is accelerated as sows consumed more P, which may indicate that P requirements are met.

*
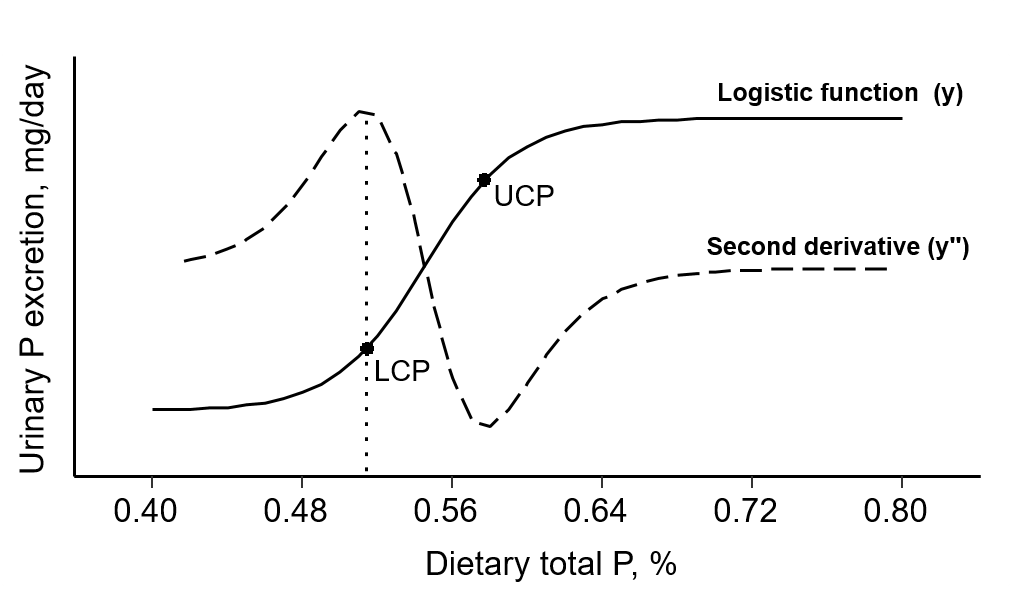
*

**Figure 1.** Illustration of the four-parameter logistic regression curve, the second derivative of the logistic function, and critical points used to estimate total P requirements in gestating sows. The lower critical point (LCP) was defined as the estimated total P requirement (%). UCP, upper critical points.

**References**

1. Gahl MJ, Finke MD, Crenshaw TD, *et al.* (1991) Use of a four-parameter logistic equation to evaluate the response of growing rats to ten levels of each indispensable amino acid. *J. Nutr.* **121**, 1720–1729.

*Supplementary Table S1*. Effect of dietary total P concentration on 24-hour urine excretion (g/day) in gestating and lactating sows*

|  | Total P, % | | | | | |  | *P*-value | |
| --- | --- | --- | --- | --- | --- | --- | --- | --- | --- |
| Period | 0.40 | 0.48 | 0.56 | 0.64 | 0.72 | 0.80 | SEM | Linear | Quadratic |
| Mid gestation | 4374 | 6459 | 3597 | 4196 | 3869 | 4662 | 1861 | 0.73 | 0.81 |
| Late gestation | 5018 | 6728 | 6416 | 4120 | 9438 | 6223 | 3071 | 0.66 | 0.92 |
| Early lactation | 8175 | 7480 | 5606 | 6316 | 6240 | 7483 | 1504 | 0.65 | 0.17 |
| Late lactation | 13741 | 15237 | 9641 | 10189 | 11060 | 9219 | 2235 | 0.06 | 0.50 |
| Next gestation | 25843 | 32722 | 29490 | 30004 | 20571 | 39104 | 9283 | 0.78 | 0.77 |

* Values represent the least squares means. Parity number was used as a covariate. Data were collected on days 77.1 + 2 (mid gestation) and 112.4 + 1 (late gestation) of gestation, days 4.5 + 1 (early lactation) and 18.2 + 1 (late lactation) of lactation, and day 35.5 + 1 of the subsequent gestation (next gestation).

The amount of urine excreted (g/day) for each period of total 24-hour urine collections was not significantly affected by dietary treatments. Urine output during the first gestation (mid gestation and late gestation) was numerically lower than in the next gestation. As described in the Material and methods section, in the first gestation period sows were housed in farrowing crates from day 76.1 + 2 of gestation to facilitate urine collections. However, in the next gestation, sows were housed in gestation crates throughout the entire gestation period. The large differences in urine output between the two gestation periods may be attributable to the watering system. In gestation rooms, water was provided in troughs, whereas in lactation rooms via nipple drinkers. An additional factor might be associated with different seasons of the year when urine samples were collected for the two gestation periods. Measurements in the next gestation occurred in summer months.

*Supplementary Table S2*. Evaluation of goodness of fit of the regression models used to estimate total P requirements

| Period* | Model | Total P, %^†^ | 95% CI | R^2^_adj_^‡^ | AIC^‡^ | Total P, g/d^§^ |
| --- | --- | --- | --- | --- | --- | --- |
| Mid gestation^\|\|^ | Logistic | 0.51 | - | 0.72 | 122.2 | 10.2 |
|  |  |  |  |  |  |  |
| Late gestation^¶^ | Plateau-linear | 0.45 | 0.39 - 0.50 | 0.70 | 126.8 | 9.0 |
|  | Logistic | 0.52 | - | 0.73 | 123.6 | 10.4 |
|  |  |  |  |  |  |  |
| Early lactation | Plateau-linear | 0.51 | 0.46 - 0.55 | 0.67 | 116.3 | 31.1 |
|  | Plateau-quadratic | 0.42 | 0.38 - 0.46 | 0.66 | 116.9 | 25.6 |
|  | Logistic | 0.57 | - | 0.66 | 117.8 | 34.8 |
|  |  |  |  |  |  |  |
| Late lactation | Plateau-linear | 0.53 | 0.50 - 0.57 | 0.79 | 116.4 | 40.3 |
|  | Plateau-quadratic | 0.45 | 0.43 - 0.47 | 0.80 | 114.9 | 34.2 |
|  | Logistic | 0.57 | - | 0.78 | 118.2 | 43.3 |

CI, confidence interval; R^2^_adj_, adjusted coefficient of determination; AIC, Akaike information criterion.

* Data were collected on days 77.1 + 2 (mid gestation) and 112.4 + 1 (late gestation) of gestation, and days 4.5 + 1 (early lactation) and 18.2 + 1 (late lactation) of lactation.

† Estimated total P requirement.

‡ R^2^_adj_ and AIC included calculations to adjust for the number of parameters in the models.

§ Calculated total P requirement (g/day) using feed intake data and the estimated total P requirements (%).

|| Algorithms of the plateau-linear and plateau-quadratic regression models failed to converge.

¶ Algorithms of the plateau-quadratic regression model failed to converge.
